# FiPhA: an open-source platform for fiber photometry analysis

**DOI:** 10.1117/1.NPh.11.1.014305

**Published:** 2024-02-23

**Authors:** Matthew F. Bridge, Leslie R. Wilson, Sambit Panda, Korey D. Stevanovic, Ayland C. Letsinger, Sandra McBride, Jesse D. Cushman

**Affiliations:** a Social & Scientific Systems, Inc., a DLH Holdings Corp. Company, Durham, North Carolina, United States; b National Institute of Environmental Health Sciences, National Institutes of Health, Neurobiology Laboratory, Division of Intramural Research, Durham, North Carolina, United States

**Keywords:** fiber photometry, calcium imaging, event processing, R, Shiny

## Abstract

**Significance:**

Fiber photometry (FP) is a widely used technique in modern behavioral neuroscience, employing genetically encoded fluorescent sensors to monitor neural activity and neurotransmitter release in awake-behaving animals. However, analyzing photometry data can be both laborious and time-consuming.

**Aim:**

We propose the fiber photometry analysis (FiPhA) app, which is a general-purpose FP analysis application. The goal is to develop a pipeline suitable for a wide range of photometry approaches, including spectrally resolved, camera-based, and lock-in demodulation.

**Approach:**

FiPhA was developed using the R Shiny framework and offers interactive visualization, quality control, and batch processing functionalities in a user-friendly interface.

**Results:**

This application simplifies and streamlines the analysis process, thereby reducing labor and time requirements. It offers interactive visualizations, event-triggered average processing, powerful tools for filtering behavioral events, and quality control features.

**Conclusions:**

FiPhA is a valuable tool for behavioral neuroscientists working with discrete, event-based FP data. It addresses the challenges associated with analyzing and investigating such data, offering a robust and user-friendly solution without the complexity of having to hand-design custom analysis pipelines. This application thus helps standardize an approach to FP analysis.

## 1 Introduction

Monitoring neurotransmission in the brain of freely moving animals is important to understand how real-time neuronal activity and specific behavioral events are correlated. Identifying this relationship can help elucidate the underlying circuitry of various brain regions, and how that circuitry is manipulated by various toxins, diseases, etc. One way to monitor neurotransmission is fiber photometry (FP), which is an optical technique used for recording cellular activity by detecting bulk fluorescence signals within cell populations or brain regions.^1^ Cells are fluorescently labeled using viral vectors or transgenetic approaches either at cell bodies^2^ or within axon terminals.^3^ While FP was first developed as a tool to measure intracellular calcium,^4^^–^^6^ it has recently been used to detect the binding of specific neurotransmitters to fluorescently labeled post synaptic receptors.^7^^–^^10^ Recording within multiple brain regions simultaneously is also possible with FP due to the relatively small size of the implantable optical probes and lightweight fiber cables.^8^^,^^11^

There has been increased interest in the use of FP for neuroscience, and researchers primarily depend on lock-in demodulation systems [e.g., Tucker David Technology (TDT) photometry system] or other custom setups. These systems produce an immense amount of data, which were conventionally analyzed using custom MATLAB or Python scripts. Several open-source measurement tools and analysis packages have since been developed that operate on all major operating systems and commonly used programming languages.^12^^–^^14^ These packages helped to standardize FP analysis, but: (1) these packages are optimized for lock-in demodulation systems and have either limited or absent support for other systems, and (2) they can easily analyze stimulus triggered-events through transistor-transistor-logic (TTL) pulses (e.g., tone, light, shock, and drinking), but require additional data preprocessing to analyze experiments that have events not easily triggered through TTL pulses (e.g., freezing, wheel running, grooming, rotating, rearing, etc.).

We introduce the fiber photometry analysis (FiPhA) app, which enables researchers to analyze behavioral data and FP data within the same user interface. FiPhA makes it possible for users to import multiple data formats from many photometry systems currently available on the market. We have validated that FiPhA can analyze data from a lock-in demodulation system (TDT photometry system),^15^ a camera-based system (Plexon multi-wavelength photometry system), and a spectrally resolved system, allowing users to flexibly define fluorescent activity during events and intervals of interest. FiPhA can also easily incorporate behavioral data collected using commercially available tracking systems, with the advantage that all of these datasets require little to no preprocessing before being imported into the app. Additionally, FiPhA can not only quickly align behavioral and FP data but also offers aesthetically pleasing visualizations. FiPhA thus helps standardize an approach to FP analysis that is both robust and user-friendly.

## 2 General Overview of FiPhA

FiPhA was developed using RStudio (v2022.x) and R (v4.2.x). It uses the Shiny framework to provide user interface functionality and the Plotly package for its interactive data visualizations.

**Fig. 1:**
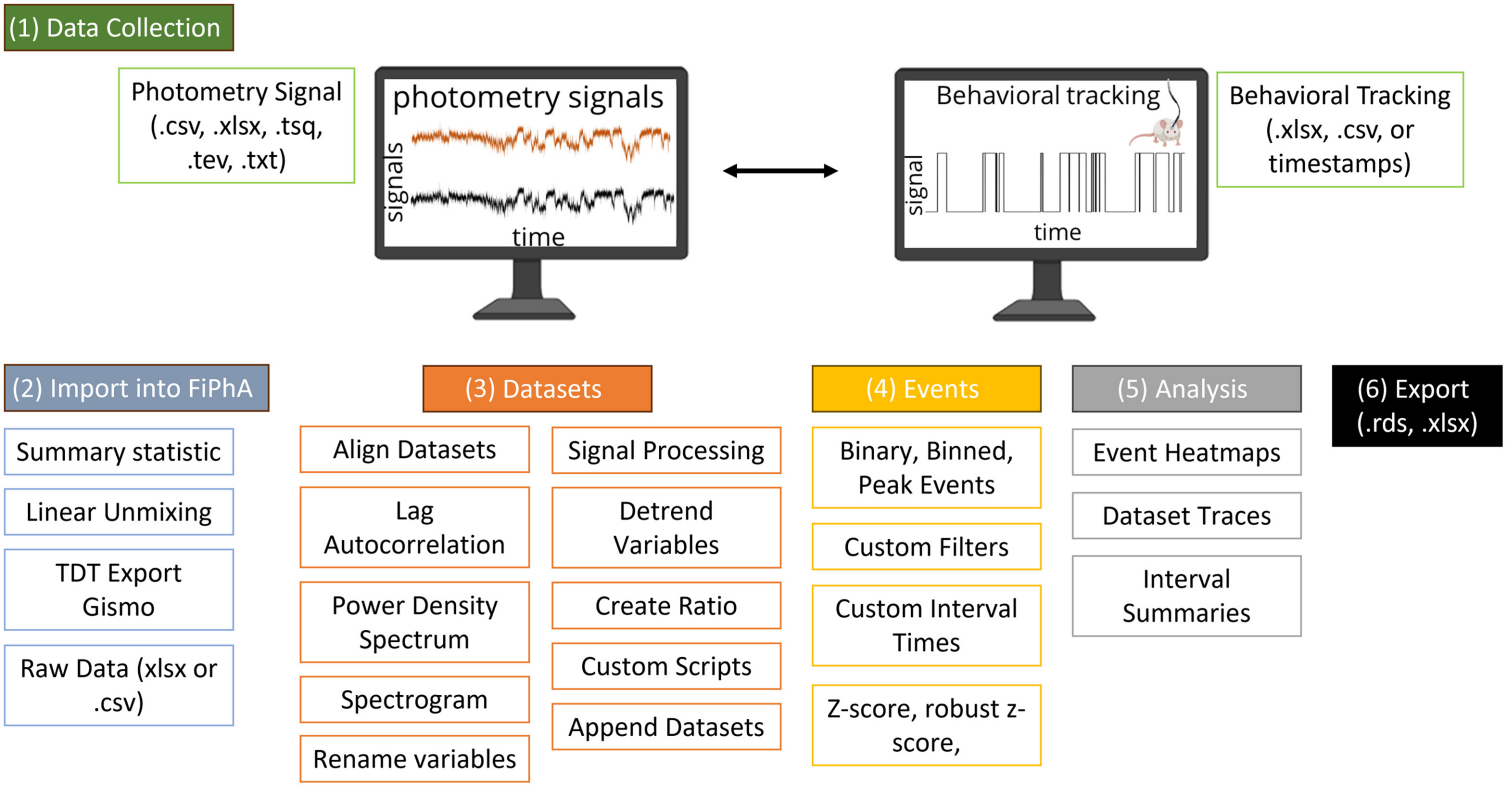
Typical workflow for analysis with the FiPhA app.

A pipeline for jointly analyzing behavioral and photometry data using the FiPhA app is illustrated in Fig. 1. This pipeline contains common steps for analyzing behavioral and photometry data in tandem, including preprocessing data, creating event-triggered averages, filtering behavioral events, and execution of various data analysis tasks. Analysis functionality includes the calculation of mean event-triggered averages, summary statistics of event intervals and their visualization in box-and-whisker plots, and graphics depicting of fluorescence activity by event across time in the form of heatmaps. The data can be saved as an “.rds” file for future analysis and the analysis of the event-triggered averages can be exported as an “.xlsx” file.

### 2.1 Datasets

#### 2.1.1 Importing recordings

During FP experiments, experimenters typically collect photometry signal and behavioral tracking data using separate software packages. The FiPhA app allows importing data from various formats (.xlsx,.csv,.txt,.tsq, etc.).

#### 2.1.2 Spectrally resolved fiber photometry import

**Fig. 2:**
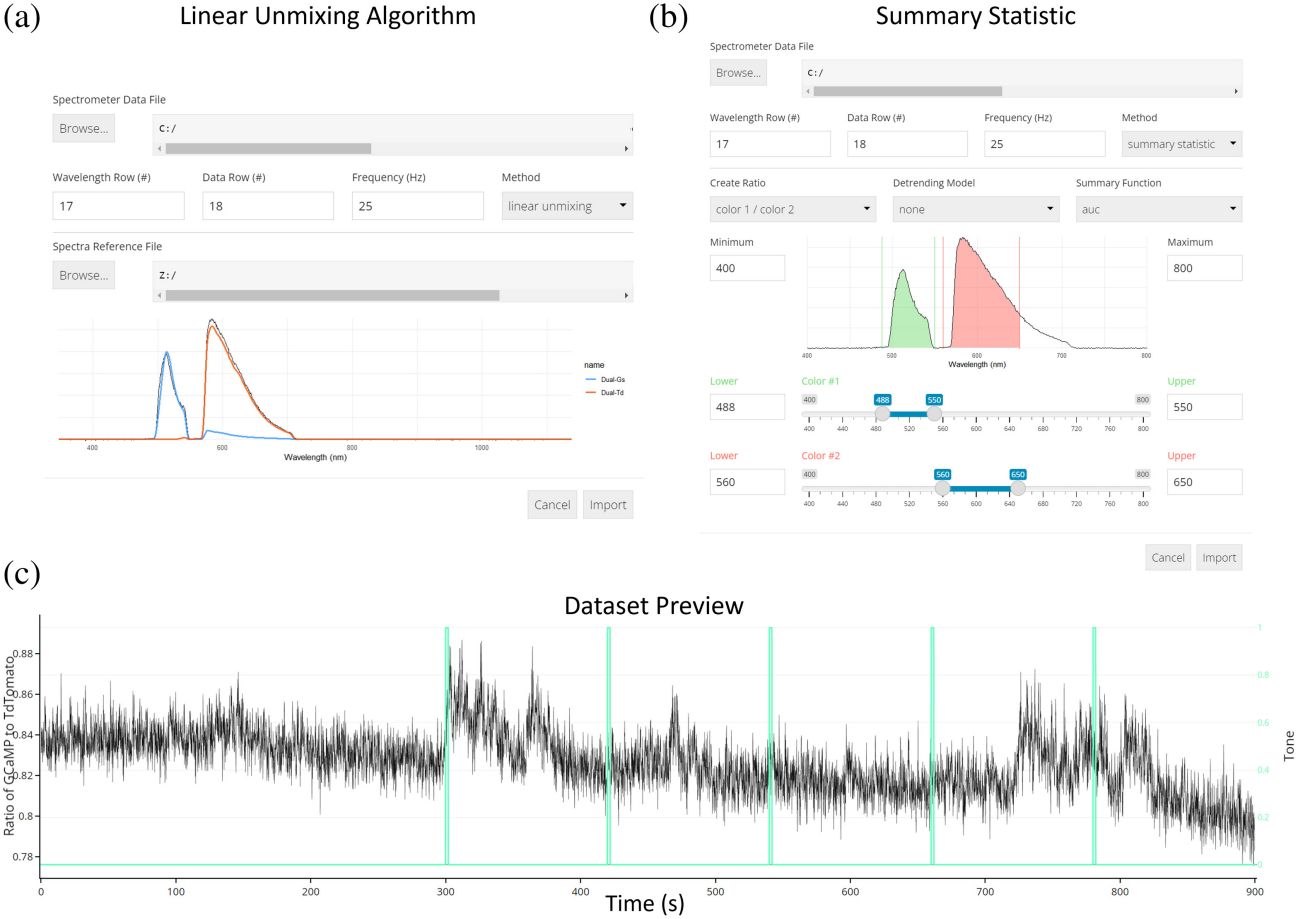
Import options for data collected using spectrally resolved photometry systems. (a) The linear unmixing algorithm showing a GCaMP and tdTomato fluorescence peak as well as a reference spectrum. (b) Importing data using the summary statistic option with calculating the area under the curve. (c) Dataset preview of a 900 s photometry recording session aligned with TTL pulses of five tone presentations.

Prior to analysis, photometry signals collected using spectrally resolved photometry systems necessarily undergo a decomposition into signals of interest through the application of either a linear unmixing algorithm or a “summary statistic”-based option with user-defined ranges of wavelengths for optimal signal identification when using fluorophores with overlapped emission spectra (Fig. 2). FiPhA simplifies the linear unmixing step by allowing import of the raw spectrometer file, set the selected wavelength row, data row, and collection frequency, and then the import of a specific spectrometer reference file within a single interface. This spectrometer reference file contains standard fluorescence spectrograms specific to the wavelengths of the experimentally collected fluorescence signals.

The “summary statistic” option allows for the creation of a ratio of two fluorescence signals derived from user-specified wavelength ranges via application of a summary function (i.e., area under the curve, mean, and median), followed by automatic signal detrending. Spectrometer and behavioral data can then be plotted in the “preview” tab located within the “datasets” view.

#### 2.1.3 Import of lock-in demodulation and camera-based photometry

Data collected from the lock-in demodulation systems (e.g., TDT photometry system) are typically saved in “.tsq” or “.tev” formats, which can be imported into the FiPhA app using the “fiber photometry Gizmo” located under the “import” tab drop-down menu. Users specify the “.tsq” or “.tev” files by selection of a target directory and the desired fluorescence channel using the “available streams” drop down menu

Some FP collection systems will save the raw data as a tabular dataset in a “.xlsx” or “.csv” format. These can be easily imported into the FiPhA app using the “tabular dataset” function under the “import” tab drop-down menu. To use this feature, the header row numbers, data row numbers, collection frequencies, and workbook sheet number will need to be specified. Data can then be similarly visualized using the “preview” tab in the “datasets” view.

Previous analysis sessions using the FiPhA app can be saved as a “.rds” file and reimported using the “previous session” import option in the drop-down menu.

#### 2.1.4 Preview

After the data are imported into the FiPhA app, the recordings can be observed in the “preview” tab. Additional recordings can then be added by simply selecting another recording to import, which then appear as a list under the top drop-down menu. In the “preview” tab, the recording time is typically depicted on the x-axis, but can be changed on the left drop-down menu. Manipulating the y-axes can be done under the right drop-down menu. Two datasets can be viewed at once using the left and right y-axes.

#### 2.1.5 Signal analysis

**Fig. 3:**
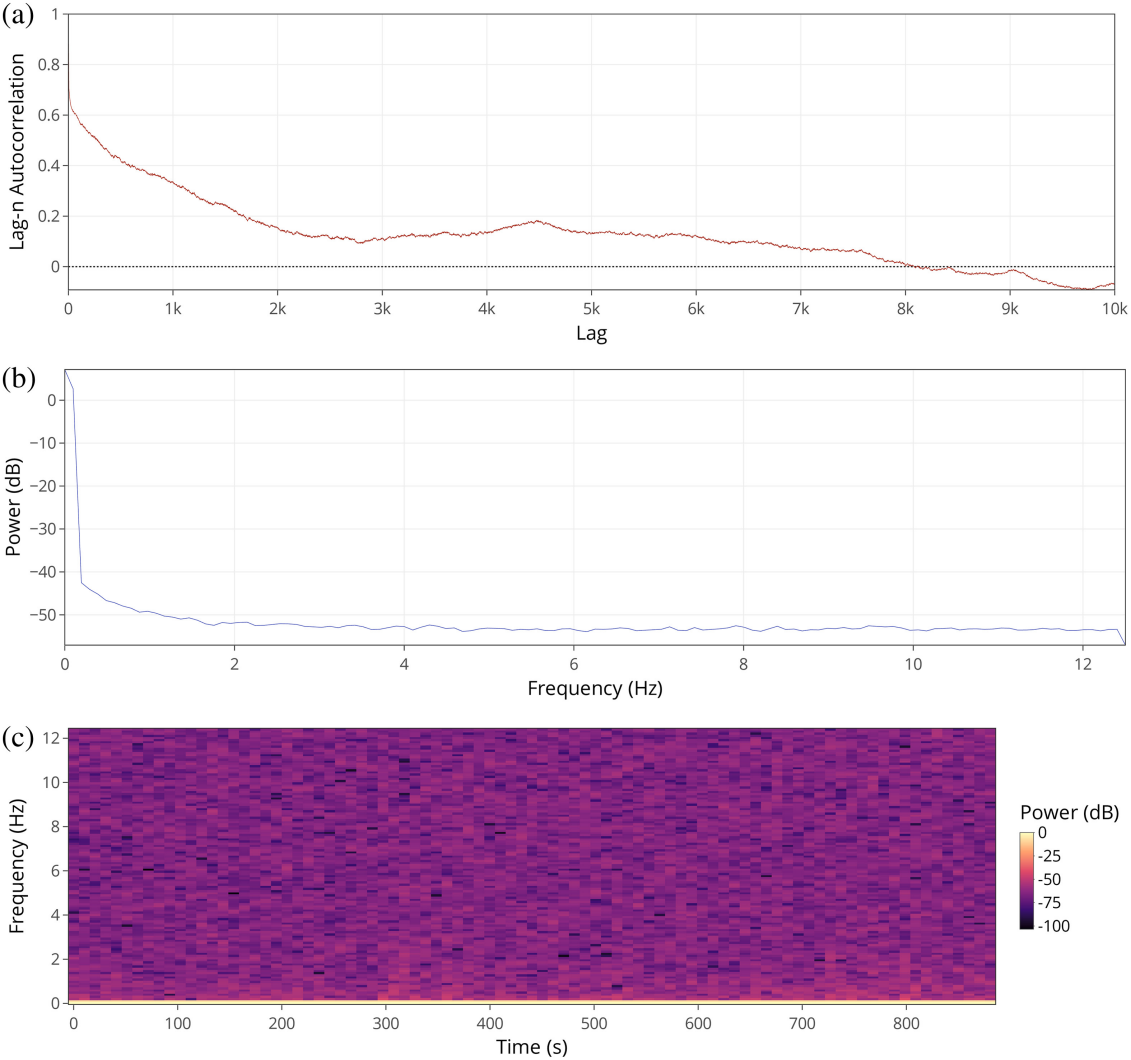
Functions located within the signal analysis tab (a), power density spectrum (b), lag autocorrelation (c), and spectrogram.

Signal analysis and investigation of periodic components of a given recording’s continuous variables can be performed using the “signal analysis” tab. Selection of a dataset will produce a lag autocorrelation plot, a power density spectrum plot, and a spectrogram along with options to customize the resulting figures (Fig. 3). Lag-N autocorrelation is a measure of a signal’s correlation with a delayed (by N time points) copy of itself, while the power density spectrum and spectrogram both visualize the overall individual frequencies that contribute to a signal and how these change over time. Although FP data is a slow signal, typically collected at 25 to 30 Hz, spectral analysis can reveal oscillatory information at or below the Nyquist frequency (approximately 12 to 15 Hz). This includes important frequencies such delta waves which are indicative of slow wave sleep.^16^

#### 2.1.6 Transforms

Recordings can be combined with additional datasets from external hardware (such as behavior information derived from video recordings) after a dataset has been imported. This functionality is available under the “transform” menu. Alignment of datasets which do not begin simultaneously or those which have been recorded at different collection frequencies are addressed by nearest neighbor sampling of the behavioral dataset and concatenation of the resulting data as a new column.

Additional operations available under the “transform” menu include the ability to append two datasets (row-wise), renaming either the dataset itself or any of its constituent variables, and calculating a ratio of two dataset variables Low-pass filters can be applied in tandem with down-sampling of larger datasets. Linear scaling transformations can also be applied, which helps adjust the magnitude of a variable to that of another and is a necessary step in recreating the “robust z-score” transformation (see Sec. 2.2.3).

**Fig. 4:**
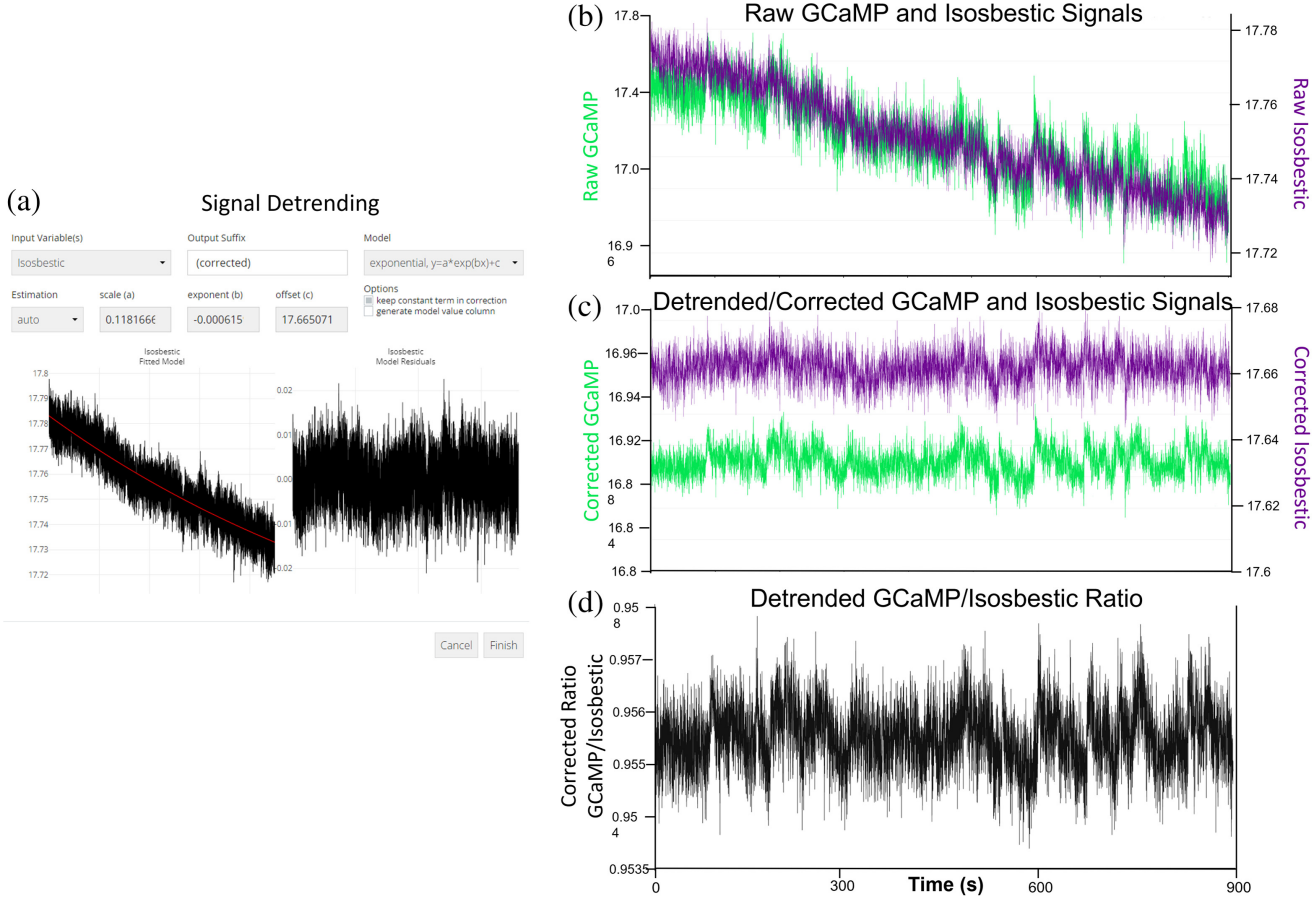
Isosbestic import and photobleaching/photoswitching corrections. (a) Detrending variables is possible using either an exponential or linear model and predicted values are provided. (b) Raw data showing two signals undergoing photobleaching/photoswitching over time. (c) The corrected channels can be visualized simultaneously in the “dataset preview.” (d) A ratio of the corrected signals can be created using the “create ratio” transformation.

Photobleaching/photoswitching is a common phenomenon seen in FP experiments whereby the magnitude of fluorescent activity decreases over time [Fig. 4(a)]. To account for this effect, activity can be modeled as a decreasing function of time via linear or exponential models under the “detrending” transformation option. Model parameters may be either manually specified or estimated using least squares fits, and model residuals; predicted values can be returned. The detrended fluorescence activity (calculated by subtracting the model’s predicted values from their raw values) can then be used for subsequent analysis. A ratio of the detrended variables can be created using the “create ratio” transformation option and the signal can be visualized in the “dataset preview” tab (Fig. 4).

#### 2.1.7 Custom scripts

Users familiar with the R programming language may optionally apply custom transformations to imported datasets before event detection. A dialog box provides a text field for users to execute snippets of code in an environment that has been set up to contain the dataset as a “data.table” object, an extension of the standard “data.frame” class in base R. Any transformations made are then carried over to the imported dataset. This functionality allows for the inclusion of complex operations in workflows that are not already features of FiPhA.

### 2.2 Events

#### 2.2.1 Binary, binned, and peak events

##### Binary events

An event series may be defined using a binary (TTL-like) indicator variable contained within a dataset. Events of this type will correspond to continuous periods which begin at each rising edge transition (0 to 1) through to the following falling edge (1 to 0). An inverted type is also available which performs the same identification strategy but instead with the logical complement of the specified variable.

##### Binned events

In some experimental setups, it may be desirable to divide a time series into equally sized bins for tasks such as peak counting. This type is parameterized by a starting time t and bin length L. Beginning at t, each successive interval of length L corresponds to a new event.

##### Peak events

Events may be identified using a peak detection method that uses a moving window of length L to identify periods of a continuous variable that exceed a user-defined threshold in terms of the number of standard deviations away from the window’s mean value.

##### Timestamped events

**Fig. 5:**
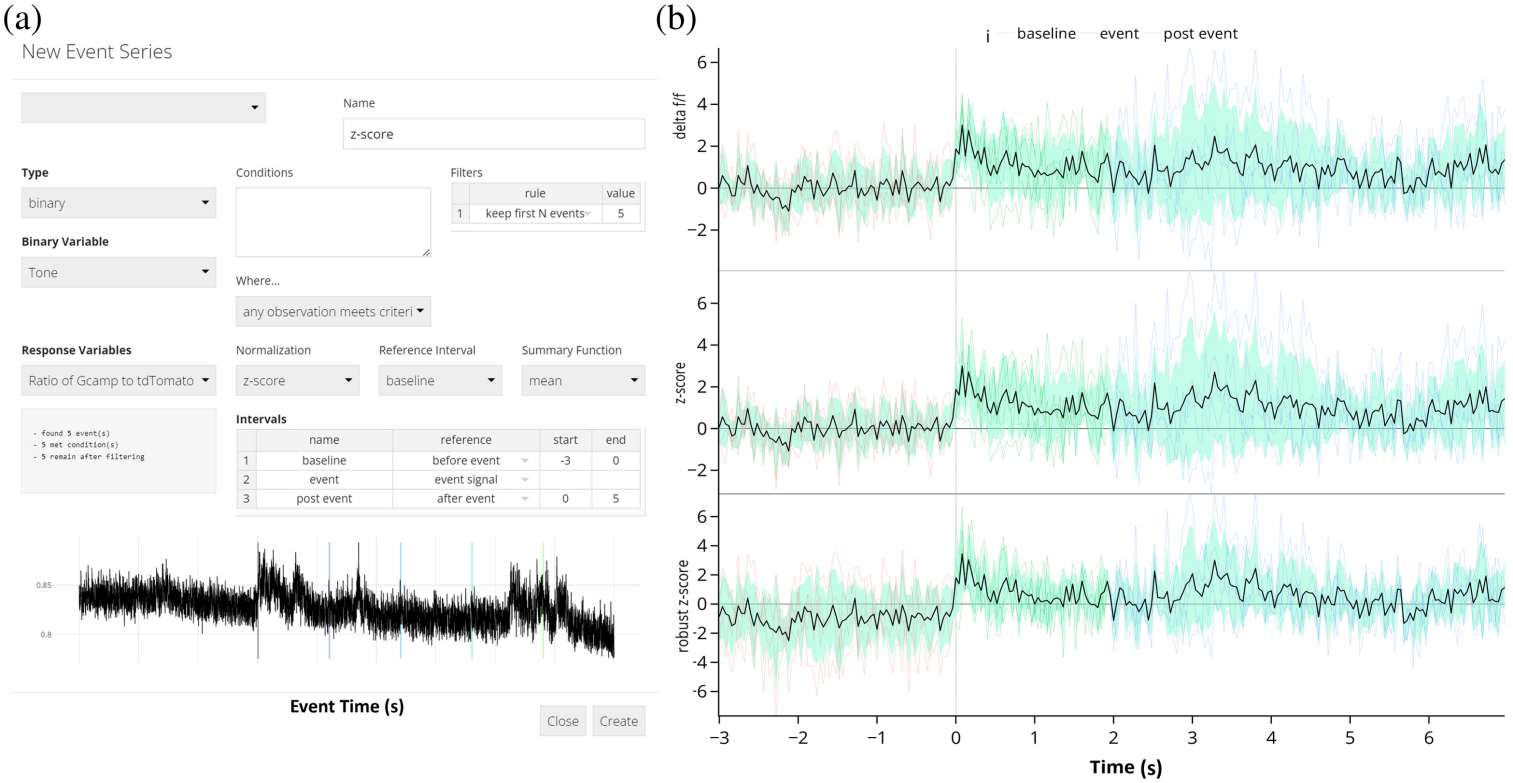
Event-triggered averages for a mouse hearing five separate tone presentations. (a) Screenshot of events window depicting events being created with a 3 s baseline and 5 s after the end of the tone. (b) Visualizations of the signal can be made using different normalization methods, such as Δ F/F, z-score, and the robust z-score. Equations for computing these quantities are in Sec. 2.2.2.

For certain experimental setups, events may not be suitably described using variables within the same dataset. In such cases, FiPhA enables users to manually input a list of start/end timestamps, which correspond to the desired events [Fig. 5(a)].

#### 2.2.2 Normalizations

The FiPhA app provides the choice of three normalization methods based on the distributional properties of the signal [Fig. 5(b)].

##### Z-score

The z-score is calculated by subtracting the mean of the interval of interest from the observation and dividing the resulting value by the standard deviation. This normalization is best suited to normally distributed, uncorrelated observations.

##### ΔF/F

This normalization is commonly used when processing FP data and is the percent change in intensity relative to a baseline period. Here, the standard deviation is replaced by the average value. Because the average may be sensitive to large values, the ΔF/F normalization may not be appropriate for noisy signals.

##### Robust z-score

A more robust z-score is calculated by subtracting the median percent change in intensity of the interval of interest from the observation and dividing by the median absolute deviation.^12^ This method is less sensitive to outlying observations than the standard z-score, but may be difficult to interpret since its reference period is no longer centered at zero.

**Fig. 6:**
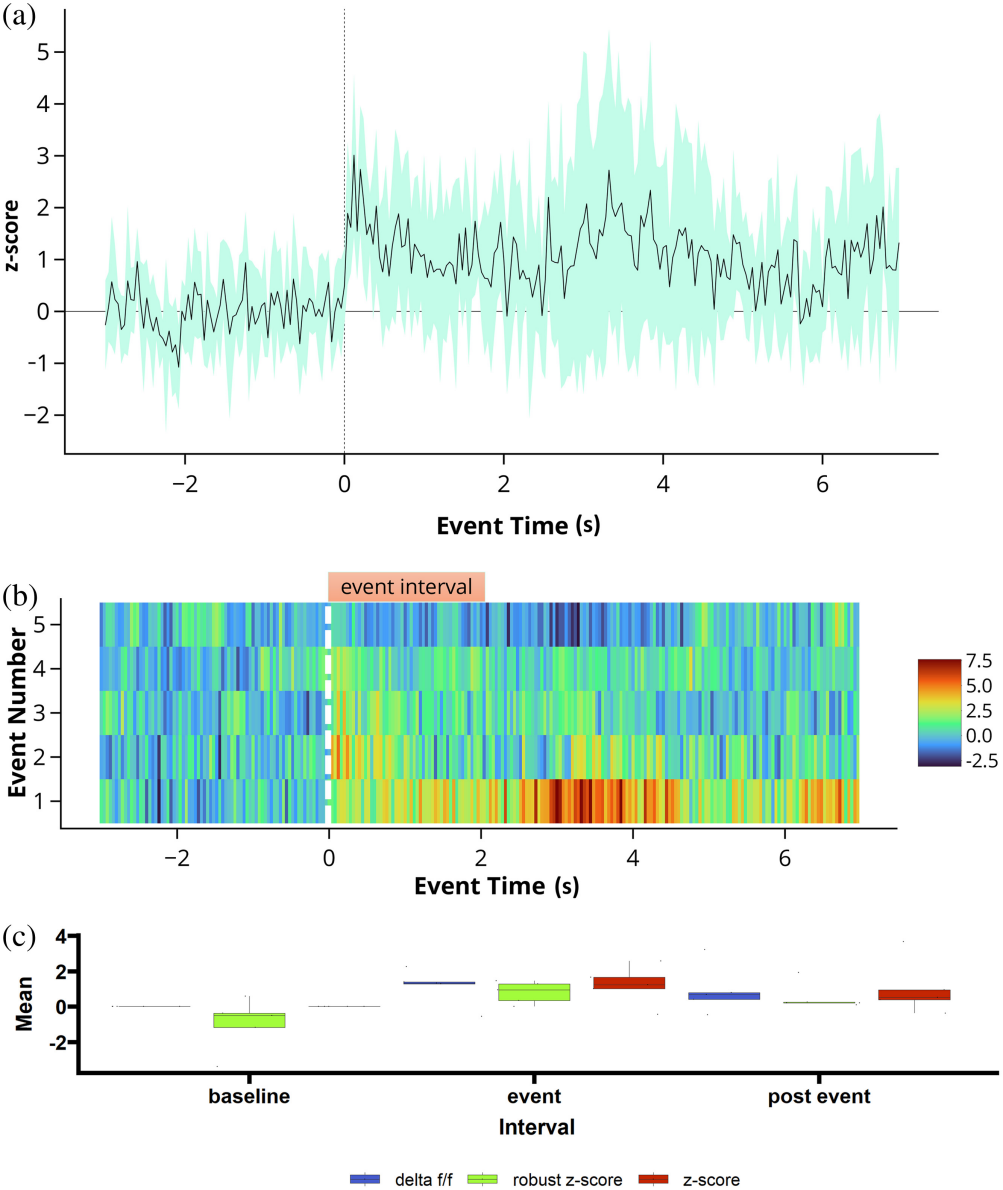
Data analysis options for viewing event-triggered averages. (a) Normalized z-score of the data traces of all event-triggered averages calculated during five tone presentations. (b) Event heatmap of same five tone presentation. (c) Interval summaries depicting box and whisker plots of the mean values of all events for each interval and normalization scheme.

#### 2.2.3 Custom filters

##### Conditions

Potential events may be conditioned on other variables within a dataset by specifying a valid R expression (e.g., ‘(time)’ < 360 & cond == T) that returns a logical value. This expression is evaluated within the context of the dataset for each time step, controlling the state of inclusion for any potential events prior to filtering. Any valid R function may also be used in the statement, and an option is available to control whether a potential event must meet the specified conditions over its entire length, or more loosely at one or more points within its length.

##### Filters

An event series definition may also include additional filters, which successively modifies the list of potential events depending on the signal type. Spurious events introduced by noisy measurements (such as those derived from video recordings of animal behavior) may be removed using some combination of these filters.

• Exclusion/inclusion of the first or last events.
• Temporal shifting of events by a constant time.
• Padding of events such that they meet a minimum length.
• Restriction of events to a maximum length of time.
• Exclusion of events that do not meet a specified minimum length.
• Exclusion of events that exceed a maximum length.
• Aggregation of events which occur within a specified amount of time of one another.
• Coalescing of overlapping events.
• Inclusion based on a minimum rate of occurrence.
• Exclusion/inclusion of events based on occurrence before/after a given timestamp.
• Exclusion of successive events which occur within a given time of one another.

#### 2.2.4 Custom interval times

Intervals of interest can be manually specified in a table, where each entry specifies the name, reference point, and start/end times in relation to the reference point. Available choices include the beginning of the event signal, the end of the event signal, the raw event signal itself, or a “beginning” variant that ensures no overlap occurs with preceding events or their intervals by adjusting the interval’s start time if necessary.

### 2.3 Analysis

The “analysis” tab within FiPhA enables users to create several different visualizations of processed event series. Each plot highlights various features of the resulting data, allowing users to investigate the full range of events in several different contexts (Fig. 6).

#### 2.3.1 Event heat maps

Heatmaps, which visualize all processed events, are produced under the “analysis” tab. Events are aligned such that their event signals all start at time zero, with options to sort events by their order, total length, or area under the curve between either the full event or two specified points [Fig. 6(b)].

#### 2.3.2 Dataset traces

Likewise, visualizations of averaged event responses are also available using the “analysis” tab. For a given dataset, multiple event series may be plotted simultaneously with options to also include a shaded area that represents the mean plus or minus a user-provided number of standard deviations (or standard errors).

#### 2.3.3 Interval summaries

Boxplots containing summary values of event interval across multiple dataset and event series can be found under the “analysis” tab. In addition to the mean interval value, the median interval value can also be plotted, as well as the area under the curve value between two time points of each event [Fig. 6(c)].

### 2.4 Export

**Fig. 7:**
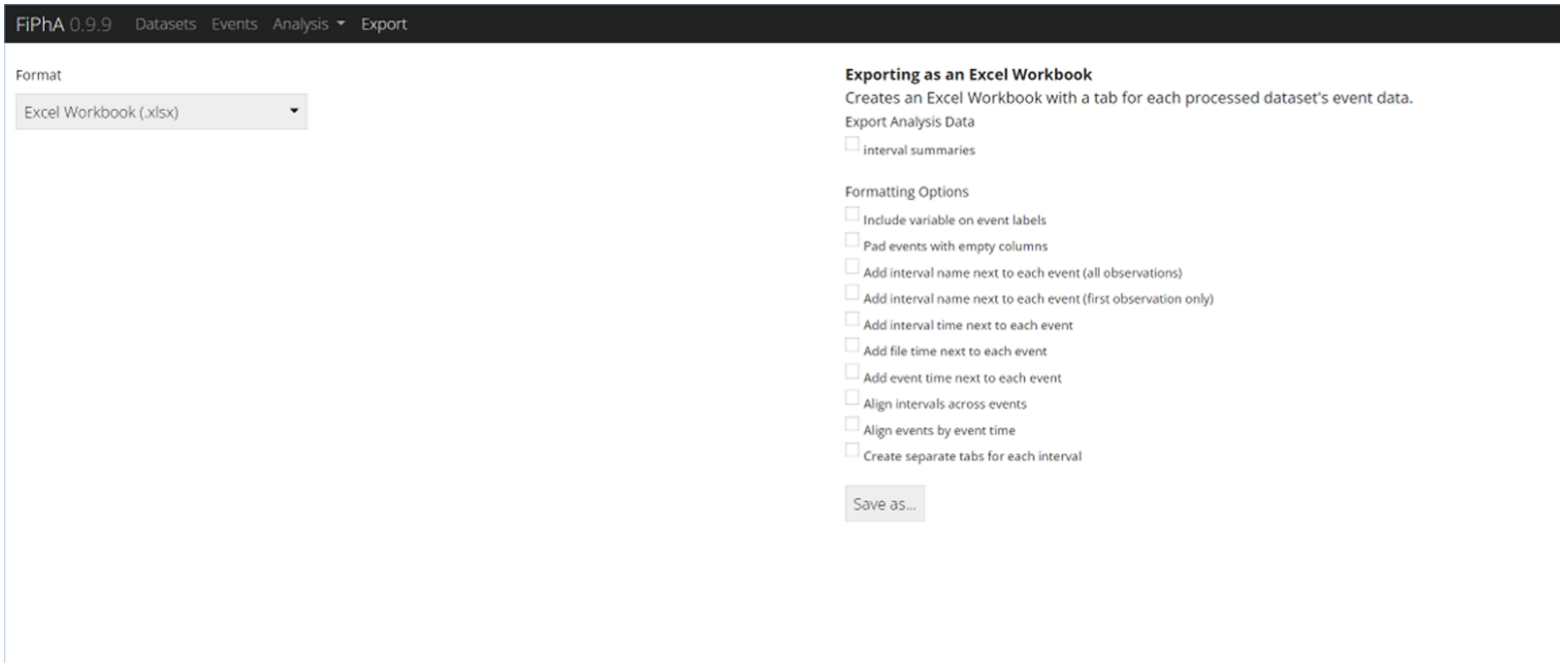
Various export options within the FiPhA app. The user can export as an excel workbook or an R data format file to be used for further analysis. The export options enable users to easily copy and paste the data into other graphing software.

The “export” tab within FiPhA allows for production of Excel workbooks, which contain all processed event data. Individual tabs corresponding to each dataset and columns for each event are included. By default, these notebooks are more machine-readable to ensure compatibility with software that deals with post-processed event data; however, several options exist to control the formatting of the resulting workbooks and produce more human-readable files. Response variable names, interval labels, and numerous timestamps (raw dataset time, time within each interval, and time relative to the start of an event signal) can also be included. Alignment of the resulting columns can also be done across events by time or interval type to assist in the comparison of events within Excel. It is also possible to save the active session as an R object, which can later be re-imported or manipulated in R without needing to use FiPhA (Fig. 7).

## 3 Example Workflow

**Fig. 8:**
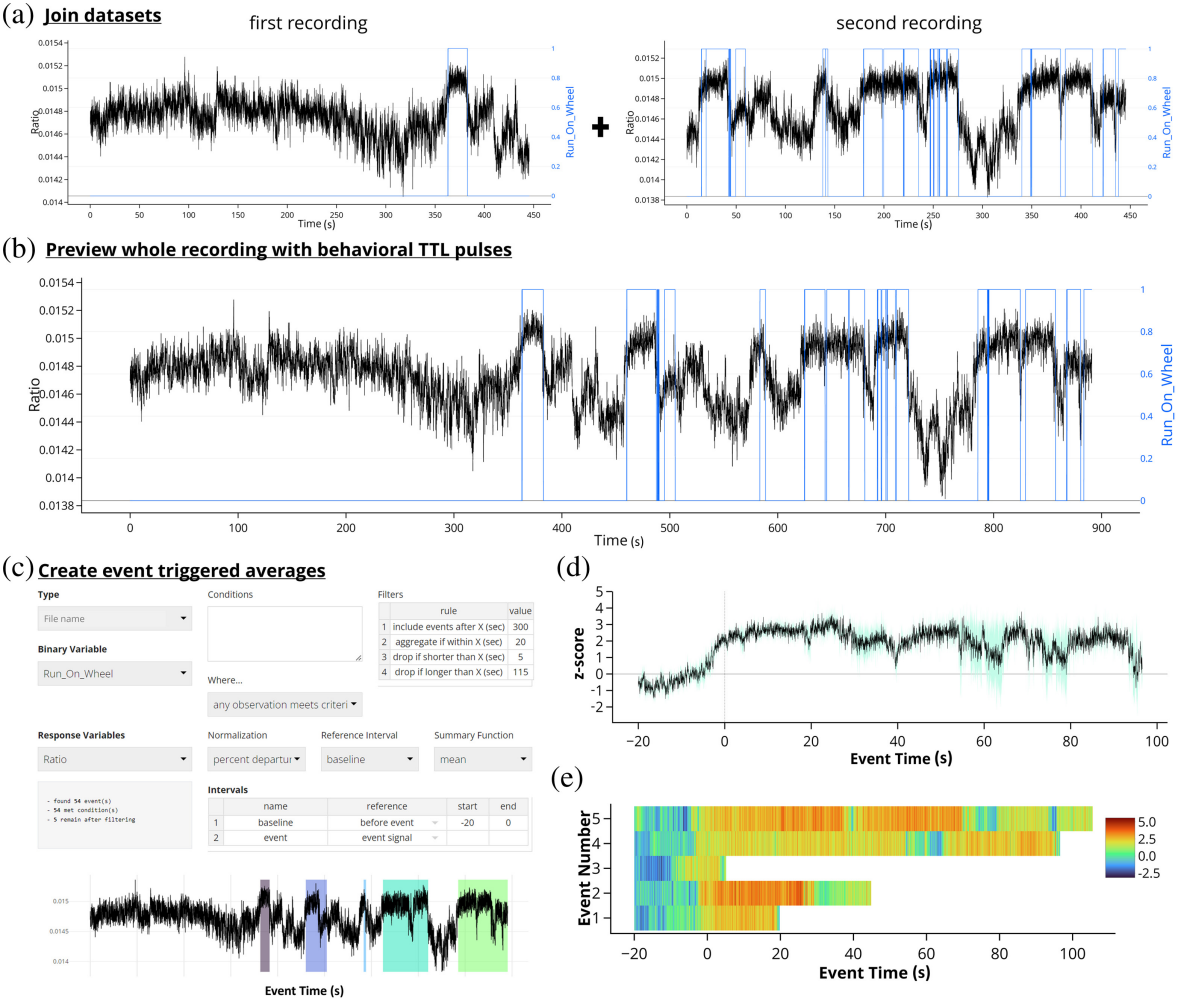
A possible workflow for computing event-triggered averages in the FiPhA app using data collected from a mouse running on a wheel before and after having a drug manipulation. (a) Behavioral tracking (blue) and photometry signal (black) were collected from two separate recordings (before and after injection) and later joined within the FiPhA app (b). (c) Events were then computed with a 20 s baseline and various filtering options, including aggregating smaller running events. Two separate graphs were then created in FiPhA by plotting (d) a normalized and averaged trace graph of all wheel-running events and (e) a heatmap of each normalized running wheel event.

The FiPhA application can analyze data without an associated TTL pulse, for example: freezing, wheel running, grooming, rotating, rearing, etc. that is derived from a synchronized video from which behavioral states are extracted. Here, an experiment was performed where ventral hippocampal acetylcholine levels during wheel running in mice was analyzed before and after receiving an intraperitoneal dose of clozapine N-oxide to alter the activity of a physical activity-related neural circuit. Two separate recordings were taken (before and after the intraperitoneal dosing) and wheel running analysis was scored using EthoVision XT 16 [Fig. 8(a)] and the raw data was exported having a timestamped binary output where “0” would be off the wheel and “1” would depict times running on the wheel. With FiPhA, photometry recordings from the ventral hippocampal acetylcholine levels can be aligned to the timestamped binary behavioral output, even if the collection frequencies are different from the two datasets. Additionally, these two separate recordings containing both the behavioral and photometric recordings can be combined and jointly analyzed [Fig. 8(b)]. From here, event triggered averages can be computed, where individual events are filtered, and interval lengths specified [Fig. 8(c)]. This experiment uses behaviorally triggered events, unlike data from previous figures which examined stimuli presented by the experimenter, so there are various lengths for each wheel running event. The ΔF/F normalization was then performed, and two different views of the event triggered averages were visualized: (1) a trace of the normalized average and deviation across all events [Fig. 8(d)] and (2) a chronological heatmap of each normalized wheel running event over time [Fig. 8(e)]. This provides an example of the flexibility with which FiPhA can be used for event-triggered averaging for behaviorally triggered events.

## 4 Discussion and Conclusion

Overall, FiPhA is a flexible and multi-purpose FP analysis tool that is broadly useful to the field. It is compatible with a broad range of photometry system data types, provides powerful visualization tools and enables customizable event definition and filtering. It is fully open source and developed to meet the needs of the community. While FiPhA was initially developed to provide an integrated approach for spectrally resolved FP, we have put extensive effort in to making it broadly applicable to various photometry methodologies and data formats. Although, not demonstrated in this manuscript, it will also be useful as the field moves increasingly toward multi-site recordings as processing of multiple photometry data streams simultaneously was built-in early in the design.

Future directions include plans for a standalone R package that allows for integration of FiPhA functionality into user scripts, which do not require the interactivity of the Shiny application, as well as the release of FiPhA in an executable format that will be far more user-friendly to those unfamiliar with R and RStudio. We plan to extend features as requested by the community and provide tutorials and training to help increase uptake and usage by interested researchers. Due to its flexibility, wide variety of features, and our plans for continued development and support, we hope that that FiPhA will prove to be of broad utility to the photometry community.

## 5 Appendix: Equations and Algorithms

### 5.1 Equations

Several transformations are provided for convenience when normalizing event responses. Each baseline period is unique, and values are not pooled across events.

#### 5.1.1 Z-score

A standard z-score transformation of x relative to a baseline, r, where $\overset{¯}{r}$ is the mean of the reference interval, and $\sigma_{r}$ is its standard deviation

$$z_{i}(x)=\frac{x_{i}-\overset{¯}{r}}{\sigma_{r}}.$$

#### 5.1.2 Delta F/F

The relative change of x to a baseline, r, where $\overset{¯}{r}$ is the reference mean. This quantity is also often expressed as a percentage^12^

$$\frac{\Delta F}{F_{i}(x)}=\frac{x_{i}-\overset{¯}{r}}{\overset{¯}{r}},$$

$$\%\frac{\Delta F}{F_{i}(x)}=100\%\times \frac{x_{i}-\overset{¯}{r}}{\overset{¯}{r}}.$$

#### 5.1.3 Robust z-score

A robust z-score-like transformation is described by Bruno et al.,^12^ which is based on medians rather than means and uses the median absolute deviation as a measure of dispersion, calculated as MAD(x)=median(|x−median(x)|). Consequently, this normalization is less sensitive to baseline noise, but it is not as straightforward to interpret as baseline values become centered about their median rather than their mean

$$RZ_{i}(x)=\frac{\%\Delta F/F_{i}(x)-\text{median}(\%\Delta F/F(r))}{\mathrm{MAD}(\%\Delta F/F(r))}.$$

### 5.2 Algorithms

Processing raw spectrometer data requires a transformation that decomposes wavelengths of interest into usable time series. Two such methods are available when importing raw spectral recordings:

#### 5.2.1 Linear spectral un-mixing algorithm

As described by Meng et al.,^17^ normalized reference spectra of each signal’s peak emission is fit to every frame of spectral data, which gives sets of linear regression coefficients as a measure of the change in fluorescence intensity over time. FiPhA’s implementation supports any number of reference spectra provided in a simple comma separated value (CSV) format with a column for wavelength bins followed by a column for each signal’s normalized response over that range of wavelengths [Fig. 2(a)].

#### 5.2.2 Summary statistic method

In cases where reference spectra are not available or the response in a particular range of wavelengths is of interest, an alternative method allows users to select ranges of wavelengths and calculate summaries based on them over time, such as their mean, median, or integrated area under the curve. This is essentially a simple deconvolution of the multidimensional spectrometer readout at each time point. These values can then be used in place of the linear unmixing process to determine the activity-dependent sensor values (e.g., GCaMP) versus the control fluorophore (e.g., tdTomato) values for calculating the final signal [Fig. 2(b)].

#### 5.2.3 Animals

All procedures related to animal use were approved by the Animal Care and Use Committee of the National Institute of Environmental Health Sciences. Animal procedures were performed in accordance with the recommendations in the Guide for the Care and Use of Laboratory animals of the National Institutes of Health (NIH).

## Data Availability

The code for the package and data for the figures presented in this article is publicly available at https://github.com/mfbridge/FiPhA.
